# Active Nuclear Import of Membrane Proteins Revisited

**DOI:** 10.3390/cells4040653

**Published:** 2015-10-13

**Authors:** Justyna K. Laba, Anton Steen, Petra Popken, Alina Chernova, Bert Poolman, Liesbeth M. Veenhoff

**Affiliations:** 1 European Institute for the Biology of Ageing (ERIBA), University of Groningen, University Medical Center Groningen, Netherlands Proteomics Centre, Antonius Deusinglaan 1, Groningen 9713 AV, The Netherlands; E-Mails: j.k.laba@rug.nl (J.K.L.); a.steen@umcg.nl (A.S.); p.popken@rug.nl (P.P.); alin.chernova@gmail.com (A.C.); 2 Department of Biochemistry, University of Groningen, Nijenborgh 4, Groningen 9747 AG, The Netherlands; E-Mail: b.poolman@rug.nl; 3 Skolkovo Institute of Science and Technology (Skoltech), Novaya St. 100, Skolkovo 143025, Russia

**Keywords:** nuclear pore complex, inner nuclear membrane, nuclear envelope, nuclear transport

## Abstract

It is poorly understood how membrane proteins destined for the inner nuclear membrane pass the crowded environment of the Nuclear Pore Complex (NPC). For the *Saccharomyces cerevisiae* proteins Src1/Heh1 and Heh2, a transport mechanism was proposed where the transmembrane domains diffuse through the membrane while the extralumenal domains encoding a nuclear localization signal (NLS) and intrinsically disordered linker (L) are accompanied by transport factors and travel through the NPC. Here, we validate the proposed mechanism and explore and discuss alternative interpretations of the data. First, to disprove an interpretation where the membrane proteins become membrane embedded only after nuclear import, we present biochemical and localization data to support that the previously used, as well as newly designed reporter proteins are membrane-embedded irrespective of the presence of the sorting signals, the specific transmembrane domain (multipass or tail anchored), independent of GET, and also under conditions that the proteins are trapped in the NPC. Second, using the recently established size limit for passive diffusion of membrane proteins in yeast, and using an improved assay, we confirm active import of polytopic membrane protein with extralumenal soluble domains larger than those that can pass by diffusion on similar timescales. This reinforces that NLS-L dependent active transport is distinct from passive diffusion. Thirdly, we revisit the proposed route through the center of the NPC and conclude that the previously used trapping assay is, unfortunately, poorly suited to address the route through the NPC, and the route thus remains unresolved. Apart from the uncertainty about the route through the NPC, the data confirm active, transport factor dependent, nuclear transport of membrane-embedded mono- and polytopic membrane proteins in baker’s yeast.

## 1. Introduction

The nuclear envelope (NE), although physically connected to the endoplasmic reticulum (ER), has features and functions that are distinct from the ER membrane system (as reviewed in [1,2,3]). It consists of two membranes: the inner and outer nuclear membrane (INM and ONM, respectively) and is perforated by Nuclear Pore Complexes (NPCs). In the pores, the INM and ONM are connected via the highly curved pore membrane. The NPCs architecture is conserved between species from yeast to higher eukaryotes, but the biology of the nuclear envelopes is distinct since yeast undergoes a closed mitosis, omitting the possibility of recruitment of INM proteins into the reassembling NE after open mitosis. In addition, the intermediate filament network of Lamin proteins underlining the INM in higher eukaryotes is not present in *Saccharomyces cerevisiae* (*S. cerevisiae*). The sorting mechanism by which membrane proteins specifically enrich at the INM may thus have conserved and species-specific aspects and is generally not well understood (reviewed in [4,5,6,7]).

The first proposed model was that metazoan INM proteins passively diffuse through the pore and are retained in the nucleus due to interactions with nuclear structures [8,9,10]. Two recent papers [11,12] detail the determinants for membrane targeting in mammalian cells using LBR (Lamin B Receptor), SUN2 (Sad1 and UNC84 domain containing 2) and LAP2β (Lamina-associated polypeptide 2β) as model substrates. The major determinants are the numbers and permeability of the NPCs, availability of binding sites at the INM, and the kinetics of diffusion through the membranes of ER. Import relies on a highly interconnected ER network, which is energy dependent [12]. Both studies [11,12] convincingly show that for these proteins a diffusion retention model of INM protein transport explains the measured kinetics of targeting in wild type and mutant cells and under conditions of energy depletion.

In the yeast *S. cerevisiae*, in addition to selective retention, an active transport route was proposed to exist for specific proteins [13,14,15,16,17]. The nuclear localization signals (NLSs) of the INM protein Heh2 and Src1/Heh1 (first described in [14]), together with an intrinsically disordered (ID) linker (L) were required and sufficient for INM accumulation of a transmembrane protein [13]. The accumulation was dependent on the transport factor Kap95 (importin-β), a functional Ran-gradient, and specific nucleoporins (Nups) rich in phenylalanine-glycine (FG) repeats (FG-Nups) and the size of the extralumenal domains [13,14]. The interpretation of this data was that the NLS and ID linker act in active transport of the transmembrane proteins through the NPC. Transport was proposed to occur through the central channel as a reporter protein could be immobilized at a central position in the NPC using affinity tags [13].

Comparing to other NLSs, the Heh1 and Heh2 NLS have structurally and biochemically distinct properties [16] that are partially shared with the NLS of Pom121 from *Rattus norvegicus* [17]. This NLS of Pom121 adapts a similar fold as the NLS of Heh1 and Heh2 when transport factor-bound and rescues the subcellular localization and synthetic sickness of Heh2ΔNLS mutants [17]. When expressed in HEK293T cells the NLS and linker of Heh2 supports INM localization. The conserved features of the NLSs of ScHeh1, ScHeh2 and RnPom121, and the effective sorting of Heh2-derived reporters in human cells, suggest that active import is conserved and confined to a subset of INM proteins.

A large part of the studies in [13,15,16,17] aimed at understanding the transport mechanism of Heh1 and Heh2 were based on studies using reporter proteins that had a C-terminal transmembrane spanning domain. A criticism to this experimental approach was that the proteins may not be in the membrane during transport and if so, in those studies effectively soluble transport was measured. This criticism is valid to some extent, as the insertion of transmembrane proteins is not fully mapped out in baker’s yeast. For years, the translocon Sec61 system in the ER was the only characterized membrane insertion/translocation machinery (reviewed in [18]), but, more recently, other systems have been described [19]. Most notably a specialized insertion system for tail-anchored proteins, called GET (Guided Entry of Tail anchored proteins), was characterized (reviewed in [20]). The function of others such as Ssh1, the non-essential Sec61 homologue in yeast [19], is still elusive. It is thus currently difficult to be certain how a specific membrane protein is inserted to the membrane and also how much redundancy exists between different systems. Most significant in this context is the question if sorting to the nucleus can precede insertion into the lipid bilayer.

Here, we present a set of experiments aimed at validating and fine-tuning the previous findings. We test if previously used Heh2-derived proteins are membrane embedded, we fine-tune and validate that active transport tolerates larger extralumenal domains than passive transport and we revisit our evidence for the transport route through the NPC.

## 2. Experimental Section

### 2.1. Strains and Plasmids

All strains and plasmids are listed in Table 1 and Table 2 of the Supplementary Material. Besides the GET deletion mutants, all the experiments described were performed in the *S. cerevisiae* K14708 strain (w303, matα tor1-1 fpr1::NAT) [21] or strains derived from it. For the construction of strains used in the NPC trapping experiments, the genes encoding nucleoporins Nup170, Nup53 and Nup59 were tagged with an FRB (FKBP12-rapamycin binding) cassette from a pFA6a-FRB-KanMX6 plasmid [22]. The strains expressing the FRB tagged Nups are viable and are normal with respect to nuclear import of cNLS-GFP. It is, however, possible that the modification of the Nups leads to rearrangements within the NPC. All the INM reporters were expressed from pACM021-GFP plasmid [13]. All the FKBP (FK506 binding protein) tagged reporters were designed based on the pJKL01-2×FKBP12-GFP-Lic-h2NLS-L-TM plasmid [13]. The plasmids encoding the Sec61 or RibU-based reporter proteins with a variable number of maltose-binding proteins (MBP) in the extralumenal domain were based on the plasmids described in [13]. In these constructs, the region encoding the transmembrane helix of Heh2 was replaced by the transmembrane domain of Sec61 or RibU, using homologous recombination.

**Table 1:** Strains.

Name	Genotype	Source
K14708	W303, Matα *tor1-1 fpr1::NAT*	[21]
Nup170FRB	K14708, *NUP170-FRB::KanMX*	This study
Nup53FRB	K14708, *NUP53-FRB::KanMX*	This study
Nup59FRB	K14708, *NUP59-FRB::KanMX*	This study
Kap95AA	K14708, *PMA1-2× FKBP12::TRP1 KAP95-FRB::KanMX*	[22]
BY4742	MATα *his3Δ1* *leu2Δ0* *lys2Δ0* *ura3Δ0*	invitrogen
GET1Δ	BY4742 *GET1::KanMX*	invitrogen
GET2Δ	BY4742 *GET2::KanMX*	invitrogen
GET3Δ	BY4742 *GET3::KanMX*	invitrogen

**Table 2:** Plasmids.

No.	Name	Description	Source
1	pACM023-G-NLS-L-TM	GFP-Heh2 (93-378) under GAL1 promoter (HIS, Cen)	[13]
2	pJKL01-F-G-NLS-L-TM	2× FKBP12 is N-terminally fused to GFP-Heh2 (93-378) from 1	[13]
3	pJKL02-PrA-F-G-NLS-L-TM	PrA is *N*-terminally fused to F-G-NLS-L-TM from 2	[13]
4	pJKL03 F-G-ΔNLS-L-TM	2× FKBP12 is N-terminally fused to Heh2 (138-378) under GAL1 promoter (HIS, Cen)	This study
5	pJKL04 F-G-NLS-ΔL-TM	As in 2, but sequence (140-302) was removed from Heh2	This study
6	pJKL05 F-G-NLS-L-TM-SN	SNAP tag is C-terminally fused to F-G-NLS-L-TM from 2	This study
7	pJKL06 G-NLS-L-TM-F	2× FKBP12 is C-terminally fused to GFP-Heh2 (93-378)	This study
8	pACM040 M-G-NLS-L-TM	As in 1, Heh2 (93-378) N-terminally fused to *MalE-GFP*	[13]
9	pACM041 M-G-M-NLS-L-TM	As in 1, Heh2 (93-378) N-terminally fused to *MalE-GFP-MalE*	[13]
10	pACM042 M-G-M-M-NLS-L-TM	As in 1, Heh2 (93-378) N-terminally fused to MalE-GFP-MalE-MalE	[13]
11	pAS12 M-G-NLS-L-Sec61TMA	As in 8, Heh2 TM1 replaced by Sec61 transmembrane domain	This study
12	pAS13 M-G-M-NLS-L-Sec61TMA	As in 9, Heh2 TM1 replaced by Sec61 transmembrane domain	This study
13	pAS14 M-G-M-M-NLS-L-Sec61TMA	As in 10, Heh2 TM1 replaced by Sec61 transmembrane domain	This study
14	pSI6 G-NLS-L-Sec61TMA	As in 1, Heh2 TM1 replaced by Sec61 transmembrane domain	This study
15	pACH1 G-NLS-L-RibU	As in 1, Heh2 TM1 replaced by RibU	This study
16	pACH2 M-G-NLS-L-RibU	As in 8, Heh2 TM1 replaced by RibU	This study
17	pACH3 M-G-M-M-NLS-L-RibU	As in 10, Heh2 TM1 replaced by RibU	This study
18	pACM045 G-NLS-L-Sec61TM1	As in 8, Heh2 TM1 replaced by Sec61 first transmembrane helix	[13]
19	pAK36	mCherry-WALP-HDEL; a fusion of mCherry to an ER marker protein under Gal1 promoter (URA, Cen)	[23]

### 2.2. Growth Conditions

Yeast strains were grown to mid exponential growth phase at 30 °C in synthetic dropout medium without L-histidine or without uracil (HDEL reporter). Strains were first grown for 1 day on medium containing 2% glucose (*w*/*v*) and then cultured on medium containing 2% raffinose (*w*/*v*). On the day of the experiment, the expression of the reporters was induced with 0.5% galactose (*w*/*v*). For the trapping experiments, rapamycin was used at a concentration of 10 ug/mL.

### 2.3. Fluorescence Microscopy

**Figure 1:**
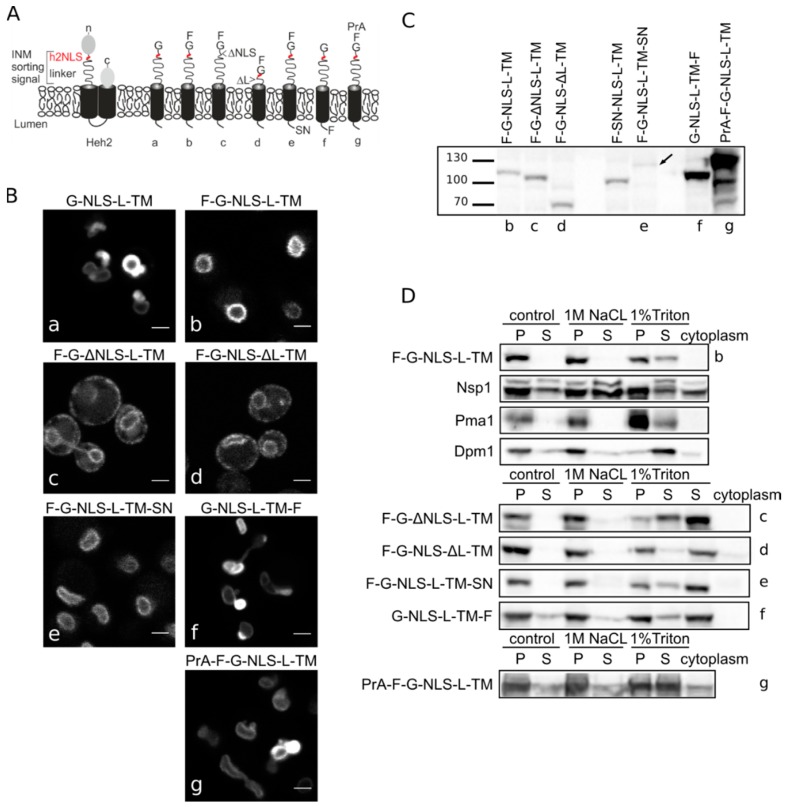
Heh2-derived reporter proteins localizing to the NE-ER network are membrane embedded. (**A**) Cartoons showing domain composition of native Heh2 and derived reporters. F: FKBP (FK506 binding protein); G: GFP; SN: SNAP-tag; PrA: ProteinA. (**B**) Confocal fluorescence microscopy images showing K14708 cells expressing the indicated Heh2-derived reporters. Scale bars: 2 μm. (**C**) Western blot (anti-FKBP) of whole cells extracts of cells expressing the indicated reporters; equal protein amounts were loaded. (**D**) Western Blots showing the results from salt and detergent extraction of crude yeast membranes fractions. Crude membranes are incubated in control buffer (20 mM Tris with 150 mM NaCl), or in buffer with 1 M NaCl or 1% Triton and ultracentrifuged, as described in Materials and Methods. The pellet (P) and supernatant (S) fractions were loaded onto the gel. The cytoplasm fraction represents the proteins present in the supernatant of the lysate after the first centrifugation step.

**Figure 2:**
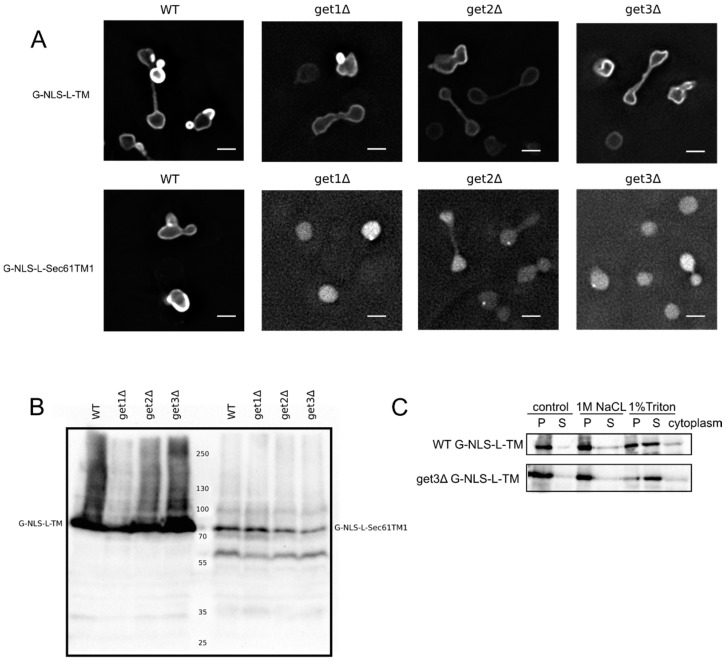
Membrane insertion of the G-NLS-L-TM reporter is GET-independent. (**A**) Fluorescence microscopy of wild type and get1Δ, get2Δ and get3Δ mutant yeasts expressing G-NLS-L-TM or G-NLS-L-Sec61TM1. Scale bars: 2 μm. (**B**) Expression levels (anti-GFP Western blot) of G-NLS-L-TM and G-NLS-L-Sec61TM1 in WT and GET mutants. (**C**) Western Blots (anti-GFP) showing the results form salt and detergent extraction assay on WT and get3Δ mutant expressing G-NLS-L-TM. Crude membrane preparations were treated as described in Material and Methods and in the legend to Figure 1. P, pellet; S, supernatant.

**Figure 3:**
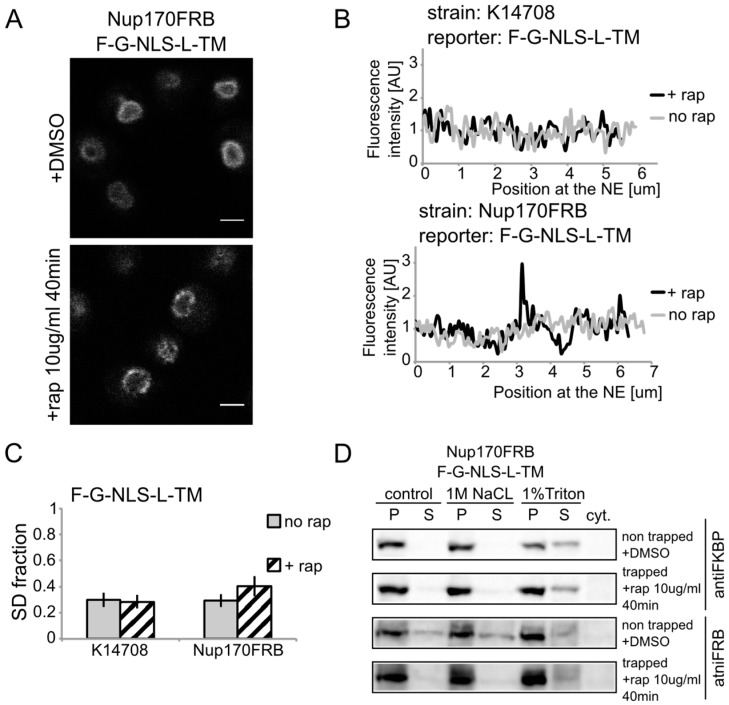
Heh2-derived reporters are membrane-embedded while transiting the NPC. (**A**) Confocal fluorescence microscopy images of cells expressing FRB (FKBP12-rapamycin binding)-tagged Nup170 (Nup170FRB) and F-G-NLS-L-TM reporter after incubation with rapamycin (+rap 10 μg/mL) and in control conditions (+DMSO). The fluorescence patterns change from continues to punctate upon rapamycin treatment. Scale bars: 2 μm. (**B**) Line-scans of the fluorescence intensity in the NE of the representative cells expressing F-G-NLS-L-TM after incubation with rapamycin (black line, +rap) or in control conditions (grey line, no rap). Top panel: control K14708 strain. Bottom panel: Nup170FRB strain. (**C**) Comparison of the average SD fraction for each reporter in the Nup170FRB strain (*n* = 37 cells for both conditions) and in the wild type (K14708, *n* = 7 cells for no rap, *n* = 55 cells for +rap); standard deviation is indicated. The SD fraction is calculated from the standard deviation of the fluorescence intensity along the NE in a cell divided by the average fluorescence intensity at the NE in that cell. (**D**) Western Blots showing the results from salt and detergent extraction of crude yeast membranes fractions of cells expressing Nup170FRB and F-G-NLS-L-TM. Membrane extractions were performed in trapped (+rap) and in non-trapped (+DMSO) conditions. F-G-NLS-L-TM solubilizes with the buffer with 1% Triton both in trapped and non-trapped conditions. Nup170FRB is salt-soluble before trapping and becomes salt-resistant upon trapping as the bands of solubilized fractions treated with control buffer and 1 M NaCl disappear.

**Figure 4:**
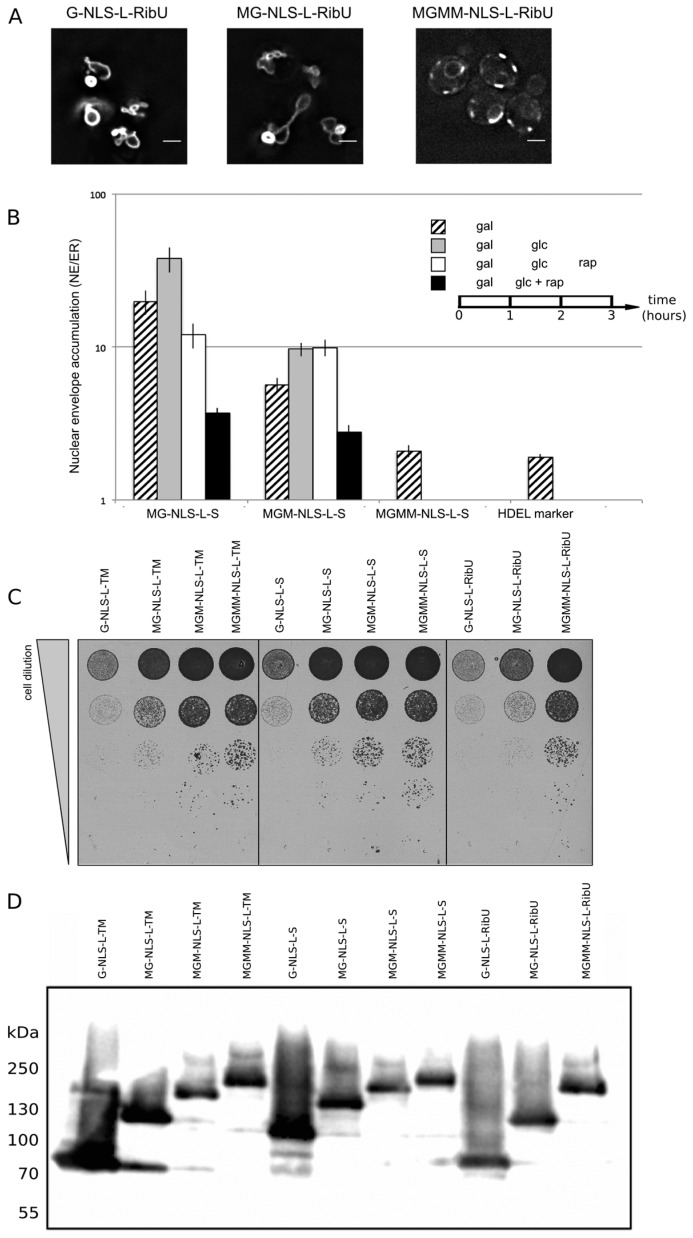
NE accumulation of membrane protein reporters with extralumenal domains of increasing size. (**A**) Fluorescence microscopy of cells expressing G-NLS-L-RibU, MG-NLS-L-RibU, MGM2-NLS-L-RibU. M: MBP. (**B**) Average accumulation of reporter proteins at the NE over the ER after different regimes of expression, and import inhibition. The reporter proteins were expressed for 1 h (striped bars); subsequently expression was inhibited by glucose for 1 h (grey bars) and finally import was blocked by rapamycin for 1 h (white bars). Alternatively, transcription and import were inhibited simultaneously (black bars). Average of 20 cells; SEM are indicated. (**C**) Viability of cells expressing membrane proteins with different transmembrane domains and differently-sized extralumenal domains. (**D**) Western blot (anti-GFP) showing expression of the membrane protein transporters with different transmembrane domains and differently-sized extralumenal domains used in this figure.

**Figure 5:**
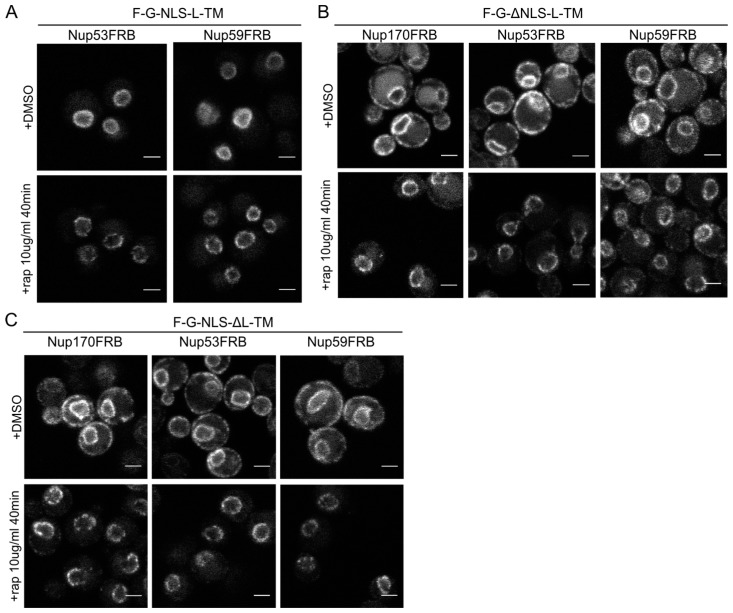
The reporters without the NLS or the linker domain can enter the NPC. (**A**) Confocal fluorescence microscopy images showing cells expressing F-G-NLS-L-TM in Nup53FRB and Nup59FRB strains background after incubation with rapamycin (+rap 10 μg/mL) and in control conditions (+DMSO). The fluorescence patterns change from continues to punctate upon rapamycin treatment (**B**,**C**). Same as (**A**), but with cells expressing F-G-ΔNLS-L-TM (**B**) or F-G-NLS-ΔL-TM (**C**) in Nup170FRB, Nup53FRB and Nup59FRB strain background. The fluorescence signal disappears from the ER upon rapamycin treatment. Scale bars: 2 μm.

The images in Figure 1, Figure 3 and Figure 5 were collected with an LSM 710 confocal microscope (CarlZeiss MicroImaging, Jena, Germany) using an objective C-Apochromat 40×/1.2NA, a solid-state laser (488 nm) for excitation and ZEN2010B software (Carl Zeiss, Jena, Germany). The images in Figure 2 and Figure 4 were collected with a Delta Vision Microscope (Applied Precision (GE), Issaquah, WA, USA), using InsightSSITM Solid State Illumination at 488 nm and an Olympus UPLS Apo 100× oil objective with 1.4 NA. Detection was done with a coolSNAP HQ2 camera (Photometrics, Tucson, AZ, USA). Image stacks (20 stacks of 0.2 um) were deconvolved using standard settings.

### 2.4. Analysis of the Standard Deviation of Fluorescence Intensity (SD Fraction Quantification)

Analysis of the fluorescent images was performed using ZEN software (ZEN2010B, Carl Zeiss, Jena, Germany) and Fiji [24]. Line-scans of the fluorescence intensity over NE were collected for at least 30 cells for each strain in each condition. For each cell, two values were calculated: average fluorescence intensity in the NE and its standard deviation. Standard deviation values for each cell were divided by the average fluorescence intensity values, and the mean value over all cells (later in this section referred to as SD fraction) was calculated from the resulting ratios. Differences in the data were considered to be significant with a *p* value less than 0.05 using a student’s *t*-test.

### 2.5. Analysis of NE/ER Ratios

Analysis was done as reported previously [15].

### 2.6. Protein Extraction and Effects of Salts and Detergents

Whole cell extracts were obtained from 10 mL cultures of exponentially growing yeast cells by lysis in NaOH and β-mercaptoethanol and TCA-precipitation. For preparation of crude membrane fractions, 200 mL of exponentially growing yeast cells were harvested by centrifugation (5500× *g* 10 min). The pellet was washed with cold demi water and centrifuged again. Subsequently, the yeast pellet was resuspended in 10 mL of 20 mM TrisHCl pH 8.0, 0.01 M EDTA with 35 uL of β-mercapthoethanol and incubated for 15 min on ice with occasional swirling. The suspension was centrifuged (rotor type: Thermo scientific SL16R 75003629, 10,000 rpm, 5 min), washed with buffer containing 1.1 M sorbitol, 50 mM TrisHCl pH 7.5, 10 mM MgCl2, 3 mM DTT and centrifuged again. The pellet was resuspended in the sorbitol buffer again to achieve 1.5 × 10^9^ cells per 1 mL. Zymolyase was added to the final concentration of 0.02%. Cells were incubated at 300 C for 1 h. The spheroplasts were pelleted (4000× *g* 10 min), carefully washed with sorbitol buffer containing 1 mM PMSF, 2 ug/mL of pepstatin and protein inhibitor cocktail and centrifuged again. From this step, the membrane isolation and solubilization of proteins with 1% Triton X-100 or 1 M NaCl were performed as described in [25]. In short, the spheroplasts were resuspended in 3 mL of 20 mM TrisHCl pH 8.0 150 mM NaCl and lysed in a 5 mL Potter tube. The cell debris was removed by short centrifugation (2 min, 2000 rpm). The supernatant was centrifuged again (20 min, 16,200× *g*) and the pellet containing membrane fraction resuspended in 500 uL 20 mM Tris HCl containing either 1% Triton X100 or 1 M NaCl or 150 mM NaCl. After 30 min incubation on ice, the samples were centrifuged (100,000× *g*, 1 h). The supernatants (solubilized fraction) and pellets (non-solubilized fraction) were mixed with SDS-PAA loading buffer, incubated at 70 °C for 10 min and loaded on SDS PAGE gels.

### 2.7. Western Blotting

Samples were separated by SDS-PAA gel electrophoresis and transferred to a PVDF membrane (wet transfer, 16 V, 17 h). For detection of the reporters, anti-FKBP antibody (Abcam, Cambridge, UK) was used at a dilution of 1:1000 (*v*/*v*) and a secondary anti-rabbit-alkaline phosphatase conjugate at 1:20,000 (*v*/*v*) (Sigma-Aldrich, St. Louis, MO, USA). For the detection of Nup170FRB, anti-FRB antibody was used at 1:1000 (*v*/*v*) (Enzo Life Sciences, Farmingdale, New York, NY, USA) and again the anti-rabbit-alkaline phosphatase conjugate. For the detection of yeast, Nsp1 anti-Nsp1 antibody was used at 1:1000 (*v*/*v*) (Abnova, Taipei, Taiwan) with anti-mouse-alkaline phosphatase conjugate at 1:20,000 (Sigma-Aldrich, St.Louis, MO, USA). For the detection of Pma1, anti-Pma1 antibody was used at 1:5000 (*v*/*v*) (Abcam, Cambridge, UK) with the anti-mouse-alkaline phosphatase conjugate. For the detection of Dpm1, anti-Dpm1 antibody was used at 1:250 (*v*/*v*) (Abcam, Cambridge, UK) with the anti-mouse-alkaline phosphatase conjugate. Pre-stained molecular weight marker (ThermoScientific, Waltham, MA, USA) was used.

## 3. Results and Discussion

### 3.1. Heh-2 Derived Reporter Proteins for Studying Import

In our previous work [13], we have defined the minimal features of the yeast *S. cerevisiae* INM protein Heh2 that govern its accumulation in the INM. In this process, Heh2 was truncated, removing the extralumenal LEM and MAN domains as well as its second transmembrane helix (TM) and lumenal domain, thereby changing it into a monotopic membrane protein. The LEM and MAN domains could cause retention in the nucleus. The resulting reporter is composed of GFP and Heh293-378 encoding the NLS, the ID linker and the first transmembrane helix, and is named G-NLS-L-TM (Figure 1A). We have previously shown that this reporter still accumulates in the INM, despite the removal of over a half of the molecular weight of native Heh2; the presence of the NLS and linker regions is required and sufficient for INM accumulation. We also showed that the accumulation is reversible upon inhibition of active transport by depletion of cytosolic Kap95 [15].

In the here presented experiments, we use the above-mentioned reporter and a reporter with an *N*-terminal FKBP tag, F-G-NLS-L-TM. Addition of the FKBP tag has a dual role—it is used in trapping experiments described later, but it also reduces the expression level of the entire protein and the NE deformation caused by high expression of INM proteins. Analysis of the microscopy data of the reporters (Figure 1B) reinstated the necessity of both the NLS and the linker region for targeting the NE as removal of the NLS (F-G-ΔNLS-L-TM) or the linker domain (F-G-NLS-ΔL-TM) abolishes NE accumulation: reporters are evenly distributed between the NE and ER. Comparison of the expression levels of all the reporters is presented in Figure 1C. They range from very low in the case of F-G-NLS-L-TM-SN (SN for SNAP tag), to very high in case of G-NLS-L-TM-F and PrA-F-G-NLS-L-TM. Higher expression levels of G-NLS-L-TM, or the reporters with C-terminal FKBP-tag (G-NLS-L-TM-F) or *N*-terminal Protein A tag (PrA-F-G-NLS-L-TM) do not interfere with accumulation at the NE, but cells do display deformation of the NE.

### 3.2. Heh-2 Derived Reporters are Integral Membrane Proteins of the NE-ER

Next, we performed biochemical fractionation studies to confirm that the reporters are membrane embedded. We measured the steady state membrane integration of the reporters by salt and detergent extraction (as published in [25]). In this method, a crude membrane fraction is isolated from exponentially growing yeast and subsequently incubated with buffers containing a high concentration of salt or detergent. After ultracentrifugation, the soluble and pellet fractions are analyzed. Proteins that solubilize with salt are only peripherally associated with the membrane, such as the nucleoporin Nsp1, which is used as a control protein. Transmembrane proteins, such as the ER protein Dpm1 with one transmembrane helix and the plasma membrane protein Pma1 with 10 transmembrane helices, require detergents to be extracted from the membrane. As shown on Western blots in Figure 1D, the integral membrane proteins solubilize only (Pma1) or predominantly (Dpm1) in 1% Triton, while the peripheral protein Nsp1 is also found in the soluble fractions when membranes were incubated with the control buffer containing 150 mM NaCl or the buffer with 1 M NaCl. The F-G-NLS-L-TM reporter behaved similarly to Dpm1 and Pma1, and solubilized in buffer with 1% Triton only. We next checked if deletion of the NLS (F-G-ΔNLS-L-TM), deletion of the linker region (F-G-NLS-ΔL-TM) or expanding the size of the soluble domains (from the cytoplasmic side PrA-F-G-NLS-L-TM and from the lumenal side F-G-NLS-L-TM-SN or G-NLS-L-TM-F) changes the solubilization pattern of these reporters. All of them solubilized predominantly or exclusively when treated with 1% Triton (Figure 1D). On the blots with G-NLS-L-TM-F and PrA-F-G-NLS-L-TM, which are the higher expressed proteins, a fraction of the protein appears in the soluble extract after incubation with the control buffer or with 1 M NaCl, similar to Dpm1. This suggests that a fraction of the reporter proteins is not well inserted in the membrane.

Altogether, the biochemical fractionation studies on whole cell lysates and the *in vivo* localization studies confirm that the majority of Heh2-derived reporter proteins are membrane embedded, and that the linker region and NLS are not critical for membrane insertion.

### 3.3. Evidence against Membrane Insertion Post Nuclear-Import

The biochemical assay presented above does not exclude if temporarily, especially during the transport via the NPC channel, a fraction of the proteins is not embedded in the membrane. This could be particularly relevant for proteins that are inserted in the membrane post-translationally and which exist shortly as a soluble, chaperoned protein. The possibility of insertion to the inner nuclear membrane after nuclear import has thus far not been tested. Many of our reporters have C-terminal transmembrane helices with a short C-terminal tail (38 residues for the G-NLS-L-TM reporter), and could thus classify as tail-anchored proteins. These types of proteins are inserted into the membrane environment post-translationally via the GET pathway [20].

We first aimed to resolve the uncertainty if our reporters depend on the GET system for membrane insertion, and tested their localization in a series of GET deletion mutants. The microscopy images and the membrane extractions with salt and detergent presented in Figure 2A,C clearly show that the G-NLS-L-TM reporter is NE localized and membrane embedded, also when the GET system is nonfunctional, *i.e.*, in a get3Δ mutant, which lacks the protein that chaperones the newly synthesized tail-anchored protein and brings it to the membrane insertion machinery. Also in get1Δ and a get2Δ mutant, lacking the proteins that are responsible for membrane insertion step of tail-anchored proteins, the reporter protein is NE localized (Figure 2A). As a control we expressed the G-NLS-L-Sec61TM1 reporter in the wildtype and GET deletion strains. In this reporter the transmembrane helix of Heh2 is replaced by the first transmembrane helix of the yeast membrane protein Sec61, and it contains a short C-terminal tail of only 10 amino acids [13]. When expressed in the wildtype yeast BY4742 the G-NLS-L-Sec61TM1 reporter localizes to the NE, in GET deletion mutants, however, this reporter localizes to the nucleoplasm and is not present in the NE (Figure 2A). Expression levels of the full length protein, and the degree of degradation of the protein, are very similar in WT and GET deletion strains (Figure 2B), showing that in the case of the G-NLS-L-Sec61TM1 reporter, membrane insertion is GET dependent and that this tail-anchored protein can be imported to the nucleus as a soluble protein when the GET insertion machinery is not functional. As we see no difference in the localization of G-NLS-L-TM between the wild type and the GET mutants, we conclude that the insertion of these reporters is not strictly dependent on GET, which would point to either Sec61-dependent co-translational insertion or by another still unidentified system.

Although chaperoned soluble nuclear import is less likely for reporters that are co-translationally inserted by Sec61, instead of post-translationally via the GET system, we aimed to further experimentally test this possibility for two reasons. Firstly, since the h2NLS-linker motif is a potent signal for sorting to the nuclear envelope outcompeting other classical NLSs [13,15,16], a competition between the membrane insertion and nuclear transport machineries might take place. Secondly, one report indicates the presence of the Sec61 machinery on the inner side of the nuclear envelope in yeast [26]. We therefore developed a system to monitor if membrane proteins were membrane embedded when they are trapped in the NPC. Our trapping experiments are based on the Anchor Away system [22], which utilizes rapamycin-dependent interaction between FRB and FKBP molecules. Previously, the FG-Nup Nsp1 had been used as the anchor in the central channel [13]. However, as recent publications show, a non-NPC cytoplasmic pool of Nsp1 [27,28], in our new set up FRB was fused to the inner ring scaffold nucleoporin Nup170.

We show rapamycin-dependent trapping of the reporter with the *N*-terminal FKBP tag, F-G-NLS-L-TM, at Nup170 (Figure 3A). After cells were exposed to rapamycin, the fluorescence signal in the NE was observed in an exclusive punctate pattern, similar to what is seen with fluorescently labeled nucleoporins. The change in fluorescence pattern is also apparent when measuring the fluorescence intensity in the NE (Figure 3B), and calculating the SD fraction (as described in the Experimental section), which represents to which degree the fluorescence on a specific location in the NE deviates from the average fluorescence intensity. The average SD fraction over multiple cells is indicated in Figure 3C. The average SD fraction increases significantly in conditions with rapamycin in the strain expressing Nup170FRB but not in the background strain (K14708), which does not have an FRB anchor at Nup170. Therefore, we conclude that a significant fraction (or possibly all) of the expressed reporter molecules were trapped at Nup170.

Using biochemical fractionation methods, we now addressed if the reporter molecules are membrane embedded when trapped in the NPC. If the reporter protein passes the NPC as a soluble protein, one expects a change in the membrane extraction characteristics (Figure 3D). When trapped at Nup170, the reporter is detergent-soluble and therefore a true transmembrane protein after entry in the pore. Consistent with this, the tight interaction with the reporter causes a change in Nup170 solubility: in non-trapped conditions Nup170 is partially extracted with salt as expected for a non-transmembrane protein, while salt-extraction is clearly prevented after rapamycin-induced binding to the reporter.

Altogether, the experiments presented in Figure 1, Figure 2 and Figure 3 show the transmembrane nature of our INM reporter proteins, also when trapped in the NPC. We find no support for a mechanism where membrane insertion occurs post nuclear import.

### 3.4. Active Transport Breaks Size Restrictions for Passive Leak through the NPC

We revisit our claim that in active import large extralumenal domains were imported; larger than what was estimated to pass the lateral channels passively. Firstly, the assays are now performed with reporter proteins with multi-pass transmembrane domain to validate that our previous studies were not biased from the use of monotopic membrane proteins. Secondly, we mapped the size limitations for passive entry of membrane proteins more precisely and established that membrane proteins with an extralumenal domain of 90 kDa still diffuse to the INM on a time-scale of an hour [29]. This now allows us to better assess if active transport indeed breaks size restrictions for passive diffusion. Thirdly, we improved the assay to better account for the impact of protein synthesis in our assay.

The new reporters consisted of the transmembrane domain of Sec61 (10 transmembrane segments). However, previous publications have suggested that Sec61 may exist in the INM [26] and thus Sec61 may have specific protein interaction partners in the INM. To exclude that the Sec61 transmembrane domain contributed to the sorting, we additionally repeated the INM targeting with reporters with a TM domain unrelated to yeast. We used the polytopic TM domain from RibU from the Gram-positive bacterium *Lactococcus lactis*, thereby excluding the chance of specific retention of the reporter in the INM via interaction with the other INM proteins. The fusion indeed sorts effectively to the INM (Figure 4A, G-NLS-L-RibU) confirming our previous claims that import is not dependent on the nature of the TM domain.

Next, a set of reporter membrane proteins with extralumenal domains of increasing size was constructed. Their extralumenal domains were composed of GFP, one, two or three copies of MBP together with the NLS-L motif, resulting in extralumenal domain size of 95 kDa (MG-NLS-L-S and MG-NLS-L-RibU); 136 kDa (MGM-NLS-L-S) and 176 kDa (MGMM-NLS-L-S and MGMM-NLS-L-RibU). From past analysis monitoring Heh2, Heh2ΔNLS [14], GFP-h2NLS-L-TM and GFP-L-TM [13] using immune electron microscopy, we know that the ratio of GFP-fluorescence in the NE and ER, (the NE/ER ratios), is a good readout of accumulation at the INM as compared to the ONM. In addition, for reporter proteins with Sec61 transmembrane domains we confirm that high NE/ER ratio’s report accumulation of the proteins at the INM as compared to the ONM (Popken *et al.*, unpublished). We thus assume that also for the here presented reporter proteins the NE/ER ratio reports localization at the INM. In the Kap95AA strain background, we find that a protein lacking complete sorting signals, such as G-ΔNLS-L-TM, gives an NE/ER ratio similar to the NE/ER ratio found for the ER-marker protein mCherry-HDEL (Figure 4B, NE/ER ratio of 1.9+/−0.1). When expressed in yeast, the MG-NLS-L-S and MGM-NLS-L-S reporters enter the nucleus and accumulate at the INM (NE/ER ratios of 19.8 and 5.7, respectively), while the MGMM-NLS-L-S reporter does not accumulate and has a NE/ER ratio of 2.1, similar to mCherry-HDEL (Figure 4B, striped bars).

To estimate how synthesis of new reporter proteins and import of already synthesized reporters affect the NE/ER ratios, we inhibited the expression of the reporters from the GAL promoter after one hour of induction, by adding glucose to the growth medium (Figure 4B, grey bars). The cultures were imaged again after one hour: the total fluorescence levels measured in the NE and the ER increased, which indicates that some reporter protein has still been synthesized and/or matured. Importantly, the accumulation levels (NE/ER) increase for the reporters MG-NLS-L-S and MGM-NLS-L-S, consistent with continued import at a reduced synthesis rates after transcription repression (Figure 4B, compare striped and grey bars). The NE/ER ratios of the MGMM-NLS-L-S and mCherry-HDEL reporters do not change in this timeframe.

Next, we determined the passive diffusion or leak of the reporters from the nucleus to the ER. For this purpose, the reporter proteins were expressed in the KAP95AA strain, which expresses Pma1-FKBP and Kap95-FRB, and in which the Kap95/Kap60-dependent active import can be conditionally blocked by the addition of rapamycin. The experiments were performed as follows: first protein expression was induced (1 h galactose), and then expression was repressed (1 h glucose), as described above. As we showed in Figure 4B, grey bars, during this hour in glucose, translation of existing mRNAs, maturation of fluorophores and import continues. Finally, rapamycin was added which results in a fast block of nuclear import [13,15,22]. The distribution of the MG-NLS-L-S and MGM-NLS-L-S reporter proteins over the NE-ER network was determined after one hour of incubation with rapamycin (Figure 4B, white bars). Only the reporter protein with the smallest extralumenal domain, MG-NLS-L-S, leaks out of the nucleus when import is blocked: the NE/ER ratio drops from 37.9 to 12.0, 1 h after addition of rapamycin (Figure 4B, compare grey and white bars). The accumulation levels of the MGM-NLS-L-S reporter do not decrease when the import is blocked, indicating that this reporter protein cannot or only very slowly passively diffuses out of the nucleus on these timescales, consistent with the size limits in [29].

When active import and transcription were inhibited simultaneously, *i.e.*, by the addition of rapamycin and glucose simultaneously after 1 h of galactose-induced expression, the NE/ER ratios decrease for both MG-NLS-L-S (from 19.8 to 3.7) and MGM-NLS-L-S (from 5.7 to 2.8) (Figure 4B, compare striped and black bars). This decrease in the NE/ER is the result of the reporter being synthesized, albeit at a reduced rate, while Kap-dependent INM import is blocked, resulting in increased fluorescence in the ER, while the NE fluorescence does not increase. The experimental scheme presented here is better than the one used previously [13,15], where we did not take into account the effect of protein synthesis in the ER.

Additional support that the type of transmembrane domain, monotopic or polytopic, is not critical for the targeting comes from viability tests. As shown in Figure 2, INM accumulation of the G-NLS-L-TM reporter results in deformed nuclei. In Figure 4 we show that the deformation correlates with the levels of INM accumulation, and also with the viability of the cells: bigger extralumenal domains result in lower accumulation (Figure 4B) and increased viability (Figure 4C). This is observed irrespective of the type of transmembrane domain, or the expression and degradation levels (Figure 4D) of the reporter proteins.

In conclusion, our findings in Figure 4 confirm that the NLS-L motif enables the import of extralumenal domains bigger than by passive diffusion. This is best illustrated by the reporter MGM-NLS-L-S; on the time scale of an hour this molecule is small enough to be accumulated at the INM by active import but it is too large to passively efflux from the NPC on this timescale.

### 3.5. Transport Route through the NPC

Previously we have shown that we could trap the same reporter as used here (F-G-NLS-L-TM) at Nsp1-FRB and concluded that the linker can span the distance between the pore membrane and the central channel. Accounting for this finding, plus the FG-Nup dependence and the large sizes of extralumenal domains that can be imported, we proposed that the extralumenal domains would travel through the center of the NPC [13]. However, recent publications show a non-NPC cytoplasmic pool of Nsp1 [27,28] that may have affected our measurements. We thus constructed new strains targeting the scaffold nucleoporin Nup170 (Figure 3), and the FG-Nups Nup53 and Nup59 as trapping sites for our reporters and tested the trapping of membrane proteins with and without the NLS-L sorting signals at these positions. All three nucleoporins are located relatively close to the pore membrane [30,31,32,33,34].

Upon incubation with rapamycin, we saw the characteristic punctate localization pattern of the F-G-NLS-L-TM reporter in all tested Nup-FRB strains (Figure 5A). The two reporters that fail to accumulate in the nuclear envelope (F-G-ΔNLS-L-TM and F-G-NLS-ΔL-TM) can also be trapped at Nup170, Nup53 and Nup59. In these cases, rapamycin does not only cause clustering of the fluorescence signal in distinct points of the nuclear envelope, but also an almost complete shift of the ER-localized molecules to the NE (Figure 5B,C). This data reinforces the mobility of our reporters between ER and the pore membrane, and also confirms that the tested reporters are not restricted from entering the NPC in the absence of a NLS or a linker region. Unfortunately, as trapping also occurs in the absence of the sorting signals, we cannot interpret the trapping event as one that uniquely reflects active import.

**Figure 6:**
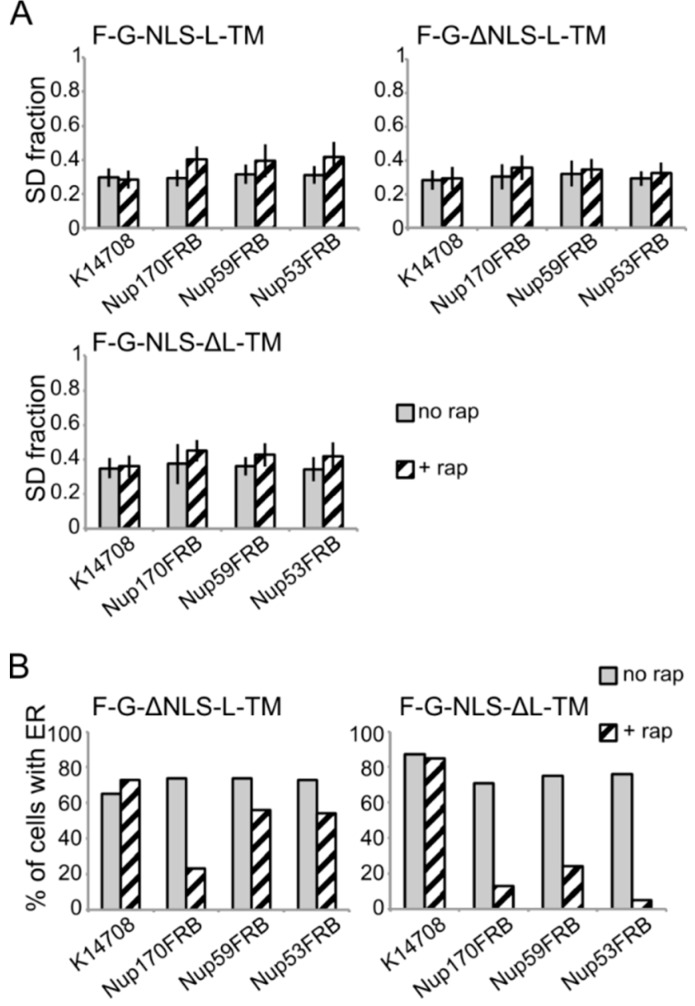
Analysis of the fluorescence images from Figure 5. (**A**) Comparison of the average SD fraction (as in Figure 3C) for F-G-NLS-L-TM, F-G-ΔNLS-L-TM, F-G-NLS-ΔL-TM in all the trap strains (Nup53FRB, Nup59FRB, Nup170FRB) and the background strain (K14708). Grey columns, control conditions (no rap); columns with black diagonal stripes, cells incubated with rapamycin (+rap). Number of cells analyzed is between 29 and 55. (**B**) Percentage of cells expressing F-G-ΔNLS-L-TM or F-G-NLS-ΔL-TM that display fluorescence in the ER in the trap strains (Nup53FRB, Nup59FRB, Nup170) and the background strain (K14708). The number of cells analyzed is between 31 and 158.

Despite the limitations of the assay, we noted differences in trapping efficiency dependent on the presence of an NLS and dependent on the trap position. The increase in SD fraction seemed smaller in the absence of the NLS (Figure 6A), possibly reflecting that trapping is less efficient. This NLS-dependent trapping efficiency is more clearly observed when looking at the percentage of cells that show fluorescence at the ER (Figure 6B). For F-G-NLS-ΔL-TM we see that the percentage of cells that show fluorescence at the ER drops from around 70% to below 20% after trapping at Nup170, Nup53 or Nup59. Trapping of the F-G-ΔNLS-L-TM reporter is less efficient at Nup59 and Nup53 as here still around 55% of the cells show fluorescence at the ER, while this value is around 20% when trapped at Nup170. The difference in trapping efficiency may reflect a different residence time of the reporters in the NPC and suggests increased residence close to Nup53 and Nup59 for reporters with the NLS as compared to those without. While the trap assay is not very suitable for dissecting the native path through the NPC, we can conclude that the extralumenal domains can reach positions in the NPC close to the membrane.

## 4. Conclusions

For the *Saccharomyces cerevisiae* proteins Src1/Heh1 and Heh2, a transport mechanism was proposed where the transmembrane domains diffuse through the membrane while the extralumenal domains encoding a nuclear localization signal (NLS) and intrinsically disordered linker (L) are accompanied by transport factors and travel through the center of the NPC. First, we investigated the membrane insertion status of the Heh2-based reporters. We found that the insertion of the reporters did not depend on the GET system, or at least was not solely dependent on it, as deletion of GET genes *GET1*, *GET2* and *GET3* did not interfere with the insertion. The Sec61 system is likely responsible for membrane insertion of our reporters although we were not able to test it directly and other systems could not be ruled out. The insertion state of our reporters was independent of the presence of the NLS, the linker or the size of luminal or extralumenal domains and depended only on the presence of a transmembrane domain. Moreover, our data pointed towards insertion in the ER preceding the transport to the NE, since we show that while (trapped) in the NPC, the reporter fractionates like an integral membrane protein. Consistently, the NPC-anchored Nup170, normally fractionating as a peripheral protein, became salt-insoluble and solely detergent soluble upon anchoring to the membrane embedded reporter protein. Altogether, these data show that our reporters do not pass the NPC in a chaperoned-soluble state but as transmembrane proteins disproving an interpretation where the membrane proteins become membrane embedded only after nuclear import.

With a new series of reporters that have the Sec61 transmembrane domain instead of a single transmembrane helix, and an improved experimental setup, we confirm that the NLS and linker domain enable the transport of proteins to the INM that would be too large for passive diffusion. The improved experimental setup takes into account that synthesis of new reporters influences the NE/ER ratio measured and reports more modest tolerance for extralumenal size than we previously reported [13]. Indeed, when simultaneously blocking nuclear transport by addition of rapamycin (which depletes the cytosol of Kap95) and transcription with glucose (which inhibits transcription of galactose-inducible reporters) one sees a decrease in the NE/ER ratio. This decrease was previously explained by efflux from the INM, but here we show that in addition residual synthesis of protein that will stay in the ER plays a role. In the current assay we inhibited the expression for one hour using glucose before addition of rapamycin. We show that with the NLS-L motif proteins having extralumenal domains bigger than 136 kDa, but smaller than 176 kDa, are actively imported. This is significantly larger than what is found for passive diffusion without the NLS-L motif, which is limited to proteins with an extralumenal domain size of approximately 90 kDa [29]. We conclude that the “NLS-L” motif enables the import of reporter proteins with extralumenal domains that are bigger than what is tolerated by passive diffusion. This is best illustrated by the reporter MGM-NLS-L-S with an extralumenal domain of 136 kDa. This protein is small enough to be imported and accumulated at the INM on the timescale of an hour but when measured on the same timescale it is too large to passively efflux from the NPC. Lastly, using yet another transmembrane domain (RibU), we re-emphasize that sorting is not dependent on the type of transmembrane domain. This set of data provides evidence that the transport route and/or the transport kinetics of membrane proteins are distinctly depending on the presence of the NLS and L motifs.

Previously, we proposed a route through the center of the NPC based on the experiments showing: FG-Nups dependence of import, the tolerance for large extralumenal domains and reporters trapping in the center of the NPC on Nsp1-FRB. In this paper, we follow up on these trap experiments and show that an extralumenal FKBP domain on the reporter could be trapped in the NPC at Nup170, Nup53 or Nup59, all positions close to the pore membrane, and even in the absence of an NLS or a linker region. These results lead us to conclude that the trap assay is unable to resolve if distinct route for passive and active transport exist. This, together with the more recently reported uncertainty of the localization of a portion of the Nsp1 pool away from the NPC [27,28], brings us to a statement that the previously reported evidence for trapping in the central channel should be considered inconclusive.

The trapping assay merely reports if the FRB and FKBP tags are ever in proximity, but the trap kinetics may resolve how rare these events are. Indeed, subtle differences in the kinetics of trapping may reflect the residence times of the FKBP tag in these reporters in the different areas of the NPC. Trapping at Nup53 and Nup59 is less efficient with the reporter lacking an NLS (F-G-ΔNLS-L-TM) compared to those with NLS-L (F-G-NLS-L-TM) or lacking the linker (G-F-NLS-ΔL-TM) while the efficiency is similar at Nup170. One explanation could be that with the NLS, the FKBP tag spends more time close to Nup53 and Nup59, which would argue that the active transport could occur close to the membrane. For a route closer to the membranes, the interaction cargo-NLS-Kap-FG may also be relevant, as FG repeats may be situated at the membrane proximity, especially at the cytoplasmic phase of the NPC. Altogether, the path through the NPC remains unresolved but at present a route closer to the pore membrane seems more likely.

The role of Kap60 and Kap95 in the localization of Heh1 and Heh2 has been debated. In the models proposed [13,14], Kap60 and Kap95 bind Heh1 or Heh2 and shuttle the proteins through the NPC. However, an alternative interpretation, that the observed Kap-dependence of Heh2 in fact reflects the Kap-dependence of a yet undefined soluble component that serves to retain the membrane protein (in an NLS-L dependent fashion) once it arrives at the INM in a Kap-independent fashion, cannot formally be excluded. We here discuss the lines of evidence in favor of a direct role for Kap60 and Kap95 in transport of Heh1 and Heh2. First, there is ample evidence for the direct and strong binding of Kap60 and Kap95 to the NLSs of Heh1 and Heh2 in both *in vivo* [15,16] and *in vitro* binding assays [13,14,16] and in structural studies [16]. Second, the importance of Kap60 and Kap95 binding for import to the INM was shown in multiple ways. For example, native Heh2-GFP or the Heh2-derived reporter are mislocalized in yeast strains carrying temperature-sensitive mutations in the Ran guanine nucleotide exchange factor or the Ran GTPase-activating protein, and in strains with temperature-sensitive mutations in KAP60 or KAP95 [13,14]. Also, natively expressed Heh2ΔNLS or point mutants of the NLS are mislocalized to the ER [14,16,17]. Moreover, the accumulation of the mobile reporter at the INM is lost upon depleting Kap95 from the cytosol (Figure 4 and previously [13,15,16,17]). Third, the accumulation of a Heh2-derived reporter at the INM is dependent on specific FG-Nups [13,14,16].

Based on these data, the most straightforward interpretation is one where the Kaps accompany the membrane proteins through the NPC. In addition, two lines of experimental evidence argue against the alternative interpretation. The first is that the mobility of the Heh2-derived reporter is not reduced at the INM compared to the ONM, which would have been expected if retained to nuclear structures [15]. Second, if the nuclear localization of the reporter proteins were based on retention to soluble nuclear components, one would expect that mutants affecting soluble transport would affect the localization of the membrane reporters in a similar way, which is not the case. For example, the accumulation of the soluble component should be reduced in the Nup188Δ strain as accumulation of cNLS-GFP reporters is reduced [35]. The accumulation of the Heh1-YFP and Heh2YFP [14] and Heh2-derived reporter (unpublished) remains, however, normal. In addition, the extent to which deletion of FG-Nups affects the transport of soluble and membrane reporter proteins is distinct [13].

Overall, the past and current studies support that in yeast Heh1 and Heh2 and the derived reporters with NLS-L motif are transported through the NPC while embedded in the membrane and are accompanied by transport factors binding the FG-Nups, and this process is energy-dependent. The spatial route through the NPC should be considered unresolved and may be more close to the membrane than previously proposed.
